# Anatomical and Physiological Responses of *Citrus* Trees to Varying Boron Availability Are Dependent on Rootstock

**DOI:** 10.3389/fpls.2016.00224

**Published:** 2016-03-04

**Authors:** Geisa L. Mesquita, Fernando C. B. Zambrosi, Francisco A. O. Tanaka, Rodrigo M. Boaretto, José A. Quaggio, Rafael V. Ribeiro, Dirceu Mattos

**Affiliations:** ^1^Centro de Citricultura Sylvio Moreira, Instituto AgronômicoCordeirópolis, Brazil; ^2^Centro de Solos e Recursos Ambientais, Instituto AgronômicoCampinas, Brazil; ^3^Departamento de Fitopatologia e Nematologia, Escola Superior de Agricultura Luiz de Queiroz, Universidade de São PauloPiracicaba, Brazil; ^4^Department of Plant Biology, Institute of Biology, University of CampinasCampinas, Brazil

**Keywords:** leaf gas exchange, growth, micronutrient, nutritional stress, microscopy, water relations

## Abstract

In *Citrus*, water, nutrient transport and thereby fruit production, are influenced among other factors, by the interaction between rootstock and boron (B) nutrition. This study aimed to investigate how B affects the anatomical structure of roots and leaves as well as leaf gas exchange in sweet orange trees grafted on two contrasting rootstocks in response to B supply. Plants grafted on Swingle citrumelo or Sunki mandarin were grown in a nutrient solution of varying B concentration (deficient, adequate, and excessive). Those grafted on Swingle were more tolerant to both B deficiency and toxicity than those on Sunki, as revealed by higher shoot and root growth. In addition, plants grafted on Sunki exhibited more severe anatomical and physiological damages under B deficiency, showing thickening of xylem cell walls and impairments in whole-plant leaf-specific hydraulic conductance and leaf CO_2_ assimilation. Our data revealed that trees grafted on Swingle sustain better growth under low B availablitlity in the root medium and still respond positively to increased B levels by combining higher B absorption and root growth as well as better organization of xylem vessels. Taken together, those traits improved water and B transport to the plant canopy. Under B toxicity, Swingle rootstock would also favor plant growth by reducing anatomical and ultrastructural damage to leaf tissue and improving water transport compared with plants grafted on Sunki. From a practical point of view, our results highlight that B management in citrus orchards shall take into account rootstock varieties, of which the Swingle rootstock was characterized by its performance on regulating anatomical and ultrastructural damages, improving water transport and limiting negative impacts of B stress conditions on plant growth.

## Introduction

Boron (B) deficiency is a widespread problem for many agricultural crops, including citrus (Shorrocks, 1997). This is mostly attributed to the fact that B is found as boric acid (H_3_BO_3_) in solution in acidic soils and is easily leached from the root zone (Kot, 2009). For this reason, B deficiency in citrus is commonly found under field conditions, where trees show stunted growth, death of stem apical meristems and consequent overgrowth of axillary buds. Additionally, malformation of vascular tissues has been reported as a long-term injury caused by severe B deficiency in fruit trees (Blevins and Lukaszewski, 1998).

On the other hand, B toxicity may occur in arid and semi-arid regions, where higher concentrations of B are expected either in ground water or arable soil layers (Papadakis et al., 2003; Bell and Dell, 2008). Under such conditions, trees show reduced vigor, delayed development and reduced fruit number and weight. Chlorotic and necrotic spots are found in older leaves under severe toxicity, with premature leaf abscision (Nable et al., 1997). In field-grown plants, crop losses arise as a consequence of excessive B uptake by plants due to non-homogenous distribution of B fertilizers (Bell and Dell, 2008; Boaretto et al., 2011).

As the permeability of the plasma membrane to B is relatively high compared with other mineral nutrients (Dordas et al., 2000; Hayes and Reid, 2004), root uptake is predominantely a non-metabolic process determined by (i) the gradient of B concentration between the soil solution and root cells, (ii) plant transpiration, (iii) formation of B complexes inside and outside of roots, and (iv) nutrient mobility in plant vessels when B in the soil solution is at adequate or excessive levels (Brown et al., 2002). Boron uptake is actively mediated when the B concentration in the soil solution is low (Tanaka and Fujiwara, 2008). Although both passive and active processes are likely present in *Citrus* (Papadakis et al., 2004; Zhou et al., 2014), the differential sensitivity of rootstocks to B stress has not been completely elucided (Wang et al., 2015).

Citrus rootstocks have been used to optimize plant growth and fruit yield and quality (Papadakis et al., 2003, 2004), as they affect water relations, mineral nutrition and the overall plant metabolism (Castle, 1995). The differencial influence of rootstocks on plant growth and fruit production may be related to root distribution, water and nutrient uptake efficiency, conducting vessels anatomy and distribution, and differences in root hydraulic conductivity (Barry et al., 2004). Such understading is critical for citrus orchard management as scions can be grafted on selected rootstock varieties to improve tolerance to abiotic stresses, such as drought and nutritional disorders (Rivero et al., 2003; Zambrosi et al., 2012; Pedroso et al., 2014; Ribeiro et al., 2014). In addition, since adequate B supply favors root growth of citrus (Boaretto et al., 2008), and thus water uptake capability of trees, B fertilization is required to improve fruit yield of orchards, particularly where droughts frequently impair flower induction and differentiation of plants. Accordingly, allocation of more adaptated rootstocks to the occurrence of B stresses, and sound field management become practical strategies to achieve sustainability in rain-fed systems.

Boron nutrition and the structural roles of this element in cell wall integrity and other physiological processes have been investigated intensely in annual grain crops (Goldbach and Wimmer, 2007). When considering perennial crops, there are few data on plant tolerance to B stresses and its association with anatomical traits of root vessels and long distance water transport (Rosolem and Leite, 2007; Fassio et al., 2009). Indeed, more comprehensive information on B-related processes is needed to improve nutritional diagnostic of plants and to create a basis for fine tuning fertilizer recommendations for field-grown trees (Wimmer and Eichert, 2013). Accordingly, this study aimed to evaluate how B availability affects the anatomical structure of roots and leaves as well as leaf gas exchange in sweet orange trees grafted on two rootstocks with contrasting horticultural performance in the field, discussing the bases of such differential performance of *Citrus* plants under B stresses.

## Materials and Methods

### Plants and Growing Conditions

The experiment was carried out over a single season under greenhouse conditions, where the average minimum and maximum air temperatures were 18 and 30°C, respectively, and air relative humidity varied from 30 to 100%. Young plants (18 months old) of sweet orange [*Citrus sinensis* (L.) Osbeck cv. Valência] grafted on Swingle citrumelo [*C. paradisi* Macf. × *Poncirus trifoliata* (L.) Raf.] – SW or Sunki mandarin [*C. sunki* (Hayata) hort. ex Tanaka] – SK rootstocks were grown in nutrient solution with varying concentrations of B to simulate root environments from B deficiency to toxicity: deficient (0.01 mg L^-1^ B), adequate (0.5 mg L^-1^ B), and excessive (5.0 mg L^-1^ B) as H_3_BO_3_.

Treatments were set up in a completely randomized design with four replications (four pots with one plant each), combining rootstocks and B concentrations. As previously reported (Boaretto et al., 2008), a B concentration of 0.5 mg L^-1^ was the most suitable for growth of young citrus trees in nutrient solution.

The plants, initially grown in a pine bark type substrate, were washed thoroughly to remove residues adhered to the root surface and transplanted into plastic pots containing 6 L of nutrient solution. Additionally, a set of three plants of each scion/rootstock combination was destructively sampled and oven-dried at 65°C to a constant weight to obtain total dry mass (DM) at the beginning of the experiment. Plant samples were ashed in a muﬄe furnace at 550°C for 3 h; ashes were dissolved in 0.1 mol L^-1^ hydrochloric acid (HCl) for total B determination using the colorimetric azomethine-H method (Wolf, 1974). Then, the total B content of plants was estimated considering the DM and B concentration (SW = 0.377 ± 0.004 mg plant^-1^ of B and SK = 0.390 ± 0.008 mg plant^-1^ of B).

Acclimation of plants to pots was achieved by growing them for 5 days in a nutrient solution without B and diluted to ¼ of ionic strength (Zambrosi et al., 2011). The ¼ strength nutrient solution was replaced by ½ of ionic strength for more 5 days and then by full-strength one [in mM: 9.6 N (11% as NH_4_^+^), 3.0 K, 4.5 Ca, 1.2 Mg, 1.2 S; and, in μM, 41.6 B, 54.0 Fe, 8.2 Mn, 2.5 Zn, and 1.0 Mo] with varying B concentrations. The electrical conductivity (EC) of the full-strength nutrient solution was approximately 1.5 dS m^-1^. Each pot was equipped with a tube extending to the bottom through which air was continuously bubbled for aeration of the nutrient solution. The pH of nutrient solution was monitored and maintained between 5.0 and 6.0 during the experimental period; the EC was also monitored. Water lost through transpiration was replaced every day with distilled water and the nutrient solution was renewed every 2 weeks.

### Leaf Gas Exchange and Plant Hydraulic Conductance

Leaf CO_2_ assimilation (*A*), transpiration (*E*), stomatal conductance (*g*_s_) and intercellular CO_2_ concentration (*C*_i_) were measured between 9:00 and 11:00 am, with an infrared gas analyzer (LI-6400, Li-Cor Biosciences, Lincoln, NE, USA) after 125 days of B treatment. The instantaneous carboxylation efficiency (*k*) was estimated as *A*/*C*_i_. Measurements were conducted on fully expanded leaves at a photosynthetic photon flux density of 800 μmol m^-2^ s^-1^ under natural variation of air temperature and humidity. Data were recorded when the total coefficient of variation was lower than 1% and there was temporal stability.

The water potential (ψ) in leaves of similar age to those used in measurements of gas exchange was measured using a pressure chamber (3005, Soil Moisture Equipment Corp., Goleta, CA, USA) at pre-dawn (6:00 am) and afternoon (2:00 pm), when *E* was also quantified using the infrared gas analyzer. Whole-plant leaf specific hydraulic conductance was estimated as *K*_L_ = E_2pm_/Δψ, with E_2pm_ evaluated at 2:00 pm and Δψ = ψ_6am_-ψ_2pm_ (Ribeiro et al., 2009).

### Microscopy Analyses

Anatomical characteristics of cells and vessels were evaluated in samples of mature leaves and roots after 130 days of B treatment. Tissue samples (20 mm^2^) observed under light microscopy were collected from the middle third of the leaf, between 8:00 and 9:00 am, fixed in Karnovsky solution (Karnovsky, 1965), dehydrated in increasing ethanol series [30, 50, 70, 90, and 100% (three times)] and then infiltrated with resin ethanol for polymerization [acrylic resin glycolmethacrylate (Leica^®^) and 100% ethanol at a ratio of 1:1 and then pure resin]. Then, blocks were cut on a microtome. Similarly, samples for SEM were fixed, dried to the critical point with CO_2_ and gold sputtered prior to observation. Details about light microscopy and scanning electron microscopy (SEM) are described in Mesquita et al. (2011).

The fixed leaf samples (Karnovsky solution) were post-fixed for 1 h with 1% osmium tetroxide, dehydrated in an increasing acetone concentration series [30, 50, 70, 90, and 100% (three times)], infiltrated and polymerized into Spurr low viscosity epoxy resin (EMS). The blocks were prepared for cutting in the ultramicrotome using a trimmer (EM Trim, Leica Microsystems Inc., Buffalo Grove, IL, USA). Sections (70 nm thick) were obtained using the ultramicrotome (Leica UC6, Leica Microsystems Inc., Buffalo Grove, IL, USA) and contrasted against uranyl acetate and lead citrate (Reynolds, 1963). Analyses were performed using a transmission electron microscope (EM900, Zeiss, Jena, Germany) equipped with a digital camera at 80 kV.

The thickness of leaf mesophyll (TLM) and root diameter (RD) were measured using the program available with the light microscope (Axiovision 4.8.3, Zeiss, Jena, Germany). Similarly, the diameter of xylem vessels (DXV) and xylem cell wall thickness (XCWT) were determined by SEM (LEO 435-VP, Cambridge Instruments, Cambridge, UK). A set of 50 cells randomly selected from 15 image slides of roots and leaves were used for each rootstock combination.

### Plant Dry Mass and Boron Accumulation

Immediately after tissue sampling for anatomical analyses, plants were separated into leaves, stems, and roots. These parts were washed in tap water, then mild detergent solution (1 mL L^-1^), and rinsed three times in deionized water. The material was dried to obtain the dry mass of leaves (LDM), stem, and roots (RDM). Plant samples were ashed for total B determination as described previously. Boron accumulation by tree parts during the experimental period (mg tree^-1^) was estimated by subtracting the total B amounts measured in young trees (before transplanting to the nutrient solution) from the total amount of B accumulated by trees at the end of the experiment.

### Data Analyses

Results were subjected to the analysis of variance in a complete factorial design with two rootstocks and three B levels. When significant effect was found (*p* < 0.05), mean values were compared by Tukey’s post test (*p* < 0.05) using the GLM procedure of the statistical package SAS^®^ (SAS Institute, 2005).

## Results

### Dry Mass Production Under Varying Boron Supply

**FIGURE 1:**
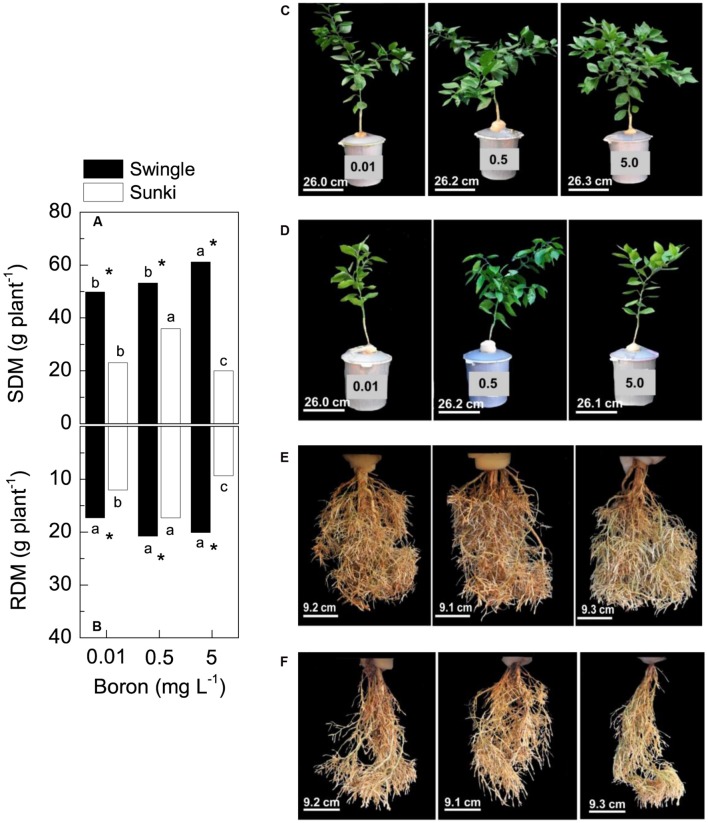
**Shoot dry mass (SDM, in A) and root dry mass (RDM, in B) of sweet orange trees grafted on Swingle (C,E) or Sunki (D,F) rootstocks grown in nutrient solution with varying concentrations of boron [C,D, in mg L^-1^, left (0.01), center (0.5), and right (5.0) columns] for 130 days.** Asterisks compare rootstocks in a given boron concentration, whereas lowercase letters indicate significant difference between boron concentrations in a given rootstock (Tukey test at *p* < 0.05).

Rootstocks and B supply affected biomass production of Valencia orange trees (**Figure 1**). For instance, trees grafted on SW were more vigorous and produced more biomass than those on SK across all B treatments (**Figures 1A,B,C,E**). The highest DM production in plants grafted on SK was found at 0.5 mg L^-1^, decreasing under either deficient (-42%) or excessive (-50%) B concentration conditions (**Figures 1A,B,D,F**). Non-significant changes in root growth due to B concentrations were observed in plants grafted on SW (**Figure 1E**).

### Plant Nutritional Status and Boron Accumulation

**FIGURE 2:**
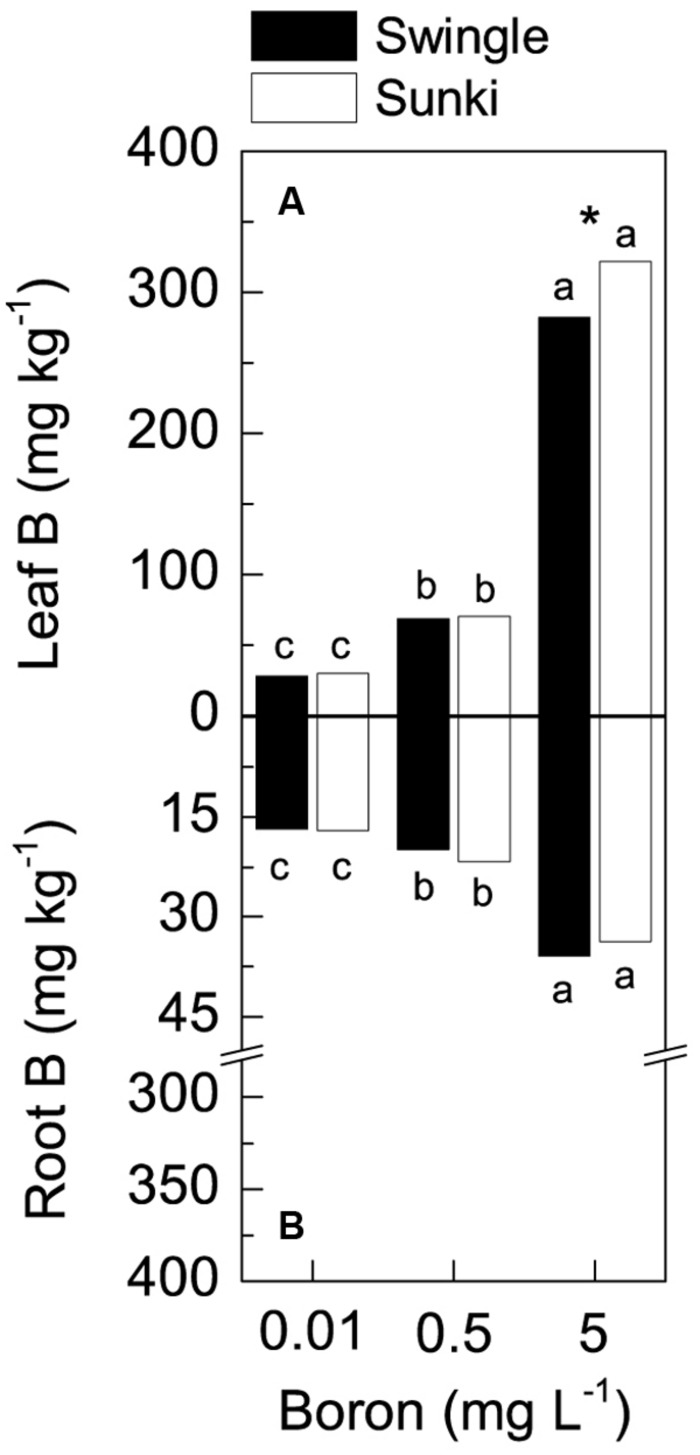
**Leaf (A) and root (B) boron concentration in sweet orange trees grafted on Swingle or Sunki rootstocks grown in nutrient solution with varying concentrations of boron for 130 days.** Asterisks compare rootstocks in a given boron concentration, whereas lowercase letters indicate significant difference between boron concentrations in a given rootstock (Tukey test at *p* < 0.05).

Leaf B concentration varied with rootstock and B treatment (**Figure 2A**). Under excessive B conditions, plants grafted on SW had lower leaf B concentrations than those on SK, whereas no differences between rootstocks were found under adequate or deficient B treatments (**Figure 2A**). Root B concentration also increased with increasing B and there was no difference between rootstocks (**Figure 2B**). Furthermore, the concentration of B in roots was much lower (up to 35 mg kg^-1^ of B) than in leaves (up to 320 mg kg^-1^ of B).

**FIGURE 3:**
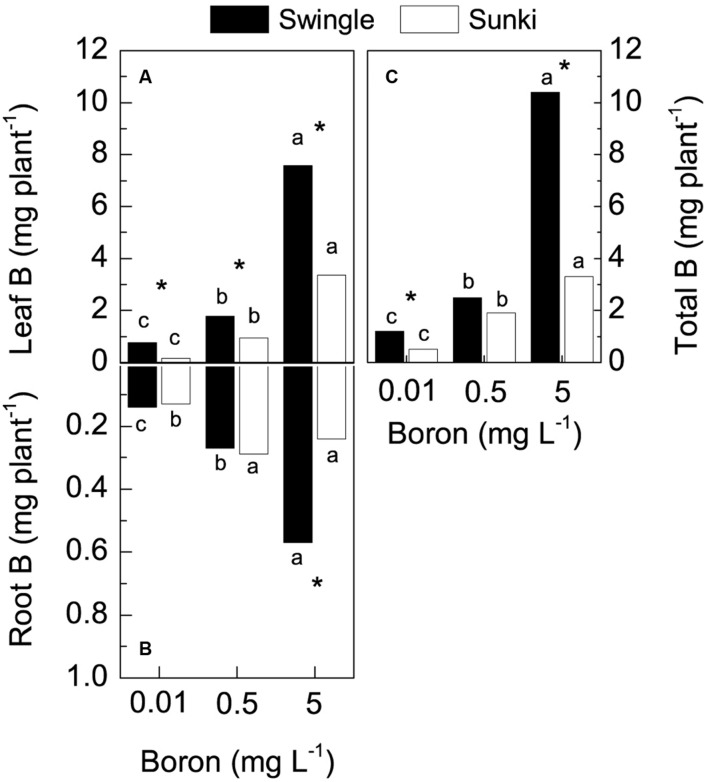
**Boron accumulation in leaves (A), roots (B), and whole plant (C) of sweet orange trees grafted on Swingle or Sunki rootstocks grown in nutrient solution with varying concentrations of boron for 130 days.** Asterisks compare rootstocks in a given boron concentration, whereas lowercase letters indicate significant difference between boron concentrations in a given rootstock (Tukey test at *p* < 0.05).

Total B accumulation also varied with treatment conditions (**Figure 3**), with plants grafted on SW exhibiting up to threefold more of the nutrient than those on SK grown under either deficiency or excess of B (**Figure 3C**). Leaves accumulated greater amounts of B in response to increased B concentration in the nutrient solution. This B accumulation was also higher in plants grafted on SW when compared to those grafted on SK (**Figure 3A**). The B accumulation in roots differed only at the highest B supply, with SW showing more than 2.4-fold the amount of B accumulated in such plant part compared with SK (**Figure 3B**).

### Physiological Traits

**FIGURE 4:**
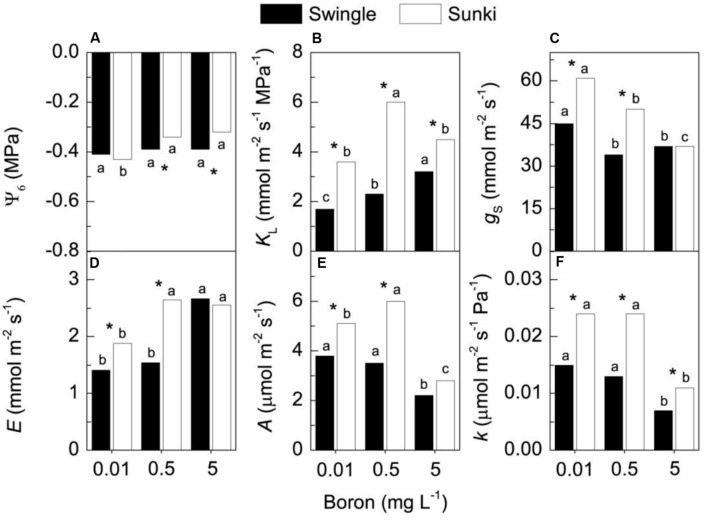
**Leaf water potential (A), whole-plant leaf specific hydraulic conductance (B), stomatal conductance (C), transpiration (D), CO_2_ assimilation (E) and instantaneous carboxylation efficiency (F) in sweet orange trees grafted on Swingle or Sunki rootstocks grown in nutrient solution with varying concentrations of boron for 130 days.** Asterisks compare rootstocks in a given boron concentration, whereas lowercase letters indicate significant difference between boron concentrations in a given rootstock (Tukey test at *p* < 0.05).

The pre-dawn leaf water potential (ψ_6am_) did not change with varying B concentration in trees grafted on SW. On the other hand, B supply increased ψ_6am_ in plants grafted on SK (**Figure 4A**). Whole-plant leaf specific hydraulic conductance (*K*_L_) increased from 0.01 to 5.0 mg L^-1^ for trees grafted on SW (**Figure 4B**), with approximately 50% change comparing the lowest and the highest B concentrations. Such a response was not noted in trees on SK, in which the highest *K*_L_ occurred at 0.5 mg L^-1^ of B (**Figure 4B**). Regardless of B supply, the *K*_L_ of plants grafted on SW was 40 to 70% lower than that of plants on SK.

Boron treatments negatively affected *g*_s_ on both rootstocks, with plants grafted on SK appearing more sensitive to increasing B supply (**Figure 4C**). SK presented higher *g*_s_ than SW at both 0.01 and 0.5 mg L^-1^. In addition to decreasing *g*_s_, B concentrations up to 5.0 mg L^-1^ increased *E* in both rootstocks (**Figure 4D**). While leaf CO_2_ assimilation (*A*) of trees on SW was not affected by increasing B from 0.01 to 0.5 mg L^-1^, it was increased by approximately 20% in plants grafted on SK (**Figure 4E**). Plants grafted on SW showed lower *A* than those grafted on SK at both 0.01 and 0.5 mg L^-1^ of B, and the excess of B caused a reduction in *A* on both rootstocks (**Figure 4E**). The highest B concentration decreased the instantaneous carboxylation efficiency (*k*), with plants grafted on SW presenting lower *k* as compared with those on SK, regardless of B treatment (**Figure 4F**).

### Structural and Ultrastructural Changes in Leaves and Roots

**FIGURE 5:**
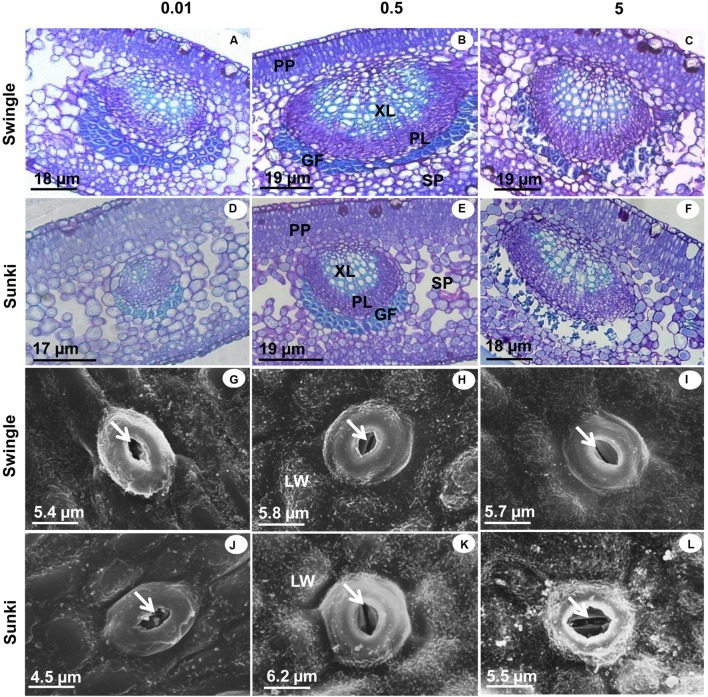
**Cross sections of leaves under light microscopy (LM) and scanning electron microscopy (SEM) of sweet orange trees grafted on Swingle (LM: A–C; SEM: G–I) or Sunki (LM: D–F; SEM: J–L) rootstocks grown in nutrient solution with varying concentrations of B for 130 days.** Concentration of nutrient solution, in mg L^-1^ of boron: left (0.01), center (0.5), and right (5.0) columns. Legend: PP, palisade parenchyma; SP, spongy parenchyma; XL, xylem; PL, phloem; GF, gelatinous fibers; LW, leaf wax; GC, guard cell; arrowhead: ostiole.

**FIGURE 6:**
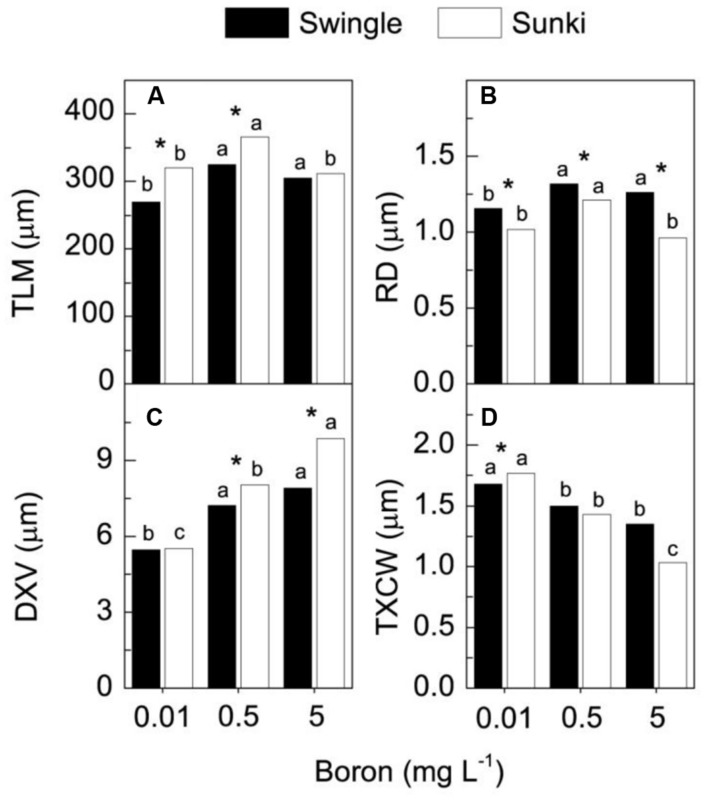
**Thickness of leaf mesophyll (TLM) (A), root diameter (RD) (B), diameter of xylem vessels (DXV) (C) and xylem cell wall thickness (TXCW) (D) in sweet orange trees grafted on Swingle or Sunki rootstocks grown in nutrient solution with varying concentrations of boron for 130 days.** Asterisks compare rootstocks in a given boron concentration, whereas lowercase letters indicate significant difference between boron concentrations in a given rootstock (Tukey test at *p* < 0.05).

Under adequate B supply (0.5 mg L^-1^), the palisade parenchyma of leaves was thinner and the spongy parenchyma cells were more regularly shaped and less spaced in trees grafted on SW than those on SK (**Figures 5B,E**). These characteristics affected the TLM, which plants grafted on SK showing significant reductions in TLM under the lowest and highest B supply (**Figures 5D,F** and **6**), compared with those grafted on SW (**Figures 5A,C** and **6**). Excessive B also caused a disturbance in compounds that form the secondary wall in SK, causing the gelatinous fibers located beneath the vascular bundle to collapse (**Figures 5D,F**). On the other hand, trees grafted on SW did not show variation in TLM when grown under either under 0.5 or 5.0 mg L^-1^ of B (**Figure 6A**).

An irregular deposition of waxes in the leaf cuticle of plants grafted on SK was apparent under 0.01 and 5.0 mg L^-1^ of B, with non-uniform covering of the leaf surface (**Figures 5J,L**): what was less evident with adequate B supply (**Figure 5K**). Structural damages in guard cells caused by varying levels of B were minimal in trees grafted on SW (**Figures 5G,H,I**) when compared with those on SK (**Figures 5J,K,L**), especially with excess B supply.

**FIGURE 7:**
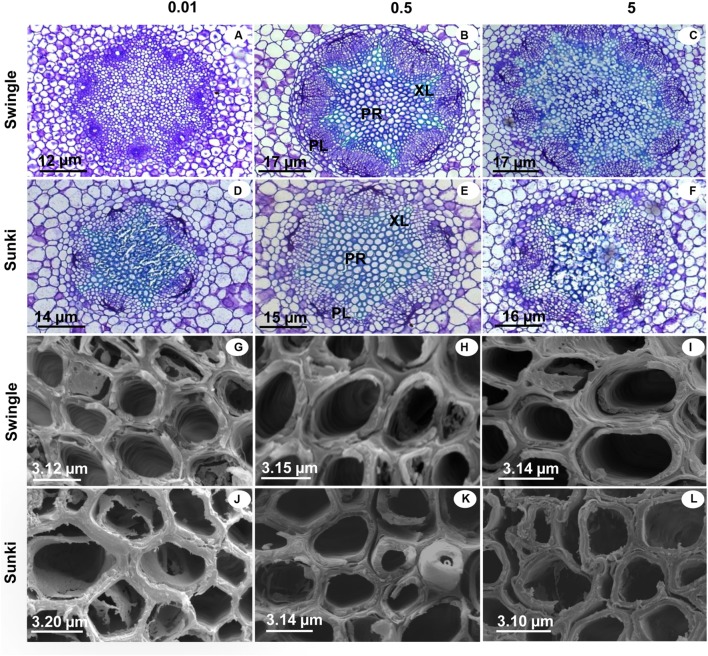
**Cross sections of roots under light microscopy (LM) and scanning electron microscopy (SEM) of sweet orange trees grafted on Swingle (LM: A–C; SEM: G–I) or Sunki (LM: D–F; SEM: J–L) rootstocks grown in nutrient solution with varying concentrations of B for 130 days.** Concentration of nutrient solution, in mg L^-1^ of boron: left (0.01), center (0.5), and right (5.0) columns. Legend: PR, parenchyma; XL, xylem; PL, phloem.

Injuries to roots were characterized by decreases in RD and in the DXV when trees were grown under low B (**Figures 6B,C**). Xylem in roots appeared spongy because of cell wall thickening (**Figures 7G,J**). Excess B did not cause significant damage to RD or DXV for plants grafted on SW (**Figures 6B,C**), whereas plants grafted on SK exhibited decreases of 20% in RD and 30% in TXCW and increase of 25% in DXV in the presence of excessive B when compared to control conditions. Furthermore, root cell walls were more curved and irregularly shaped in SK rootstock, with damaged middle lamella compromising cell linkages (**Figure 7L**).

**FIGURE 8:**
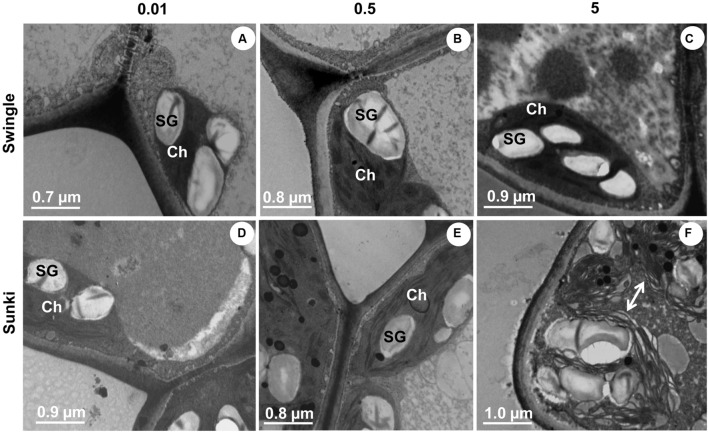
**Transmission electron microscopy of leaves of sweet orange trees grafted on Swingle (A–C) or Sunki (D–F) rootstocks grown in nutrient solution with varying concentrations of B for 130 days.** Concentration of nutrient solution, in mg L^-1^ of boron: left (0.01), center (0.5), and right (5.0) columns. Legend: SG, starch grains; Ch, chloroplast; arrow, unstructured chloroplast.

Transmission microscopy revealed that trees grafted on SK also showed significant injury to chloroplasts with disruption of thylakoids under excessive B supply (**Figure 8F**). This response was not observed in plants grafted on SW (**Figure 8C**). No clear damage was found under B deficiency (**Figures 8A,D**) or adequate supply (**Figures 8B,E**) in trees on both rootstocks.

## Discussion

As our study was carried out to evaluate the nutritional, anatomical and physiological bases of differential sensitivity of citrus rootstocks to B stress, plants were grown under B availability varying from deficiency to toxicity, conditions commonly found in many citrus growing regions around the world. Our results revealed the early responses of *Citrus* to B deficiency or toxicity before any typical plant symptoms were visible, and shed light on how rootstocks change plant sensitivity to B availability in the rooting medium. For instance, it was clearly demonstrated that plants grafted on SW exhibited greater tolerance to the B stresses conditions than those on SK, since the former rootstock was able to maintain root and shoot biomass production, even with inadequate levels of B supply (**Figure 1**).

### Physiological and Anatomical Sensitivity to Boron Deficiency

The inferior performance of plants grafted on SK under B deficiency was associated to the limited ability of this rootstock on maintaining B absorption from the nutrient solution and consequently to accumulate the nutrient (**Figure 3C**). Growth maintenance of plants depends on the continuous B supply for formation, extension, and function of meristems due to the ubiquitous role of B-rhamnogalacturonan (RG) linkages in the cell wall as well as the glycosylinositol phosphorylceramide (GIPC)-B-RG II complexes in cell membranes (Matoh et al., 1998; O’Neill et al., 2004; Hans et al., 2008; Voxeur and Fry, 2014). The argument on the influence of B absorption on the diferential ability of rooststocks to tolerate B deficiency is also supported by the fact that both rootstock varieties showed similar capacity to take up B under the adequate nutrient supply condition (0.5 mg L^-1^; **Figure 3C**). Then, the greater B accumulation seen in trees grafted on SW relative to those on SK under B deficiency was most likely favored by the combination of greater root growth (**Figure 1B,E,F**) and higher nutrient acquisition per unit of root length of the former to sustain shoot demand (Zhou et al., 2014), since trees on SW maintained higher shoot-to-root ratio (from **Figure 1**; *p* < 0.05). These findings agree with the highest B uptake ability of trees grafted on SW grown in nutrient solution labeled with ^10^B (Boaretto et al., 2008). Analysis of the expression of genes encoding B transporters will contribute to elucidate the underlying mechanisms associated with such ability of the SW root system to coordinate the expression of B transporters and channels from the external solution toward xylem vessels under varying nutrient supply (Tanaka and Fujiwara, 2008). This approach will likely provide new insights and reveal aspects of B uptake under nutrient stress conditions that have not been studied in depth in tree crops (Dannel et al., 2000; Miwa and Fujiwara, 2010).

Additionally, a better structured vascular system and greater vessel diameter found in the root system of trees grafted on SW as compared with those on SK (**Figures 6** and **7**) probably facilitated water transport and thereby B accumulation in the plant canopy on the former rootstock variety. Considering that root B concentration did not vary between rootstocks (**Figure 2B**), trees on SW were better able to use B in root tissues and maintain the well-structured xylem vessels, which would be expected if a large proportion of B could be ionically bound to pectic polysaccharides (Liu et al., 2013).

Despite the fact that SK presented a larger reduction on plant hydraulic conductance and leaf transpiration when plants were subjected to B deficiency, their values were still higher than those found on SW and no difference was detected in leaf water potential between rootstocks (**Figure 4A**). Interestingly, both rootstocks exhibited higher stomatal conductance under B deficiency compared to the adequate B supply, which was likely caused by impairment of opening and closing regulatory mechanism of guard cells under B deficiency. However, we lack clear information linking direct effects of B deficiency on stomatal apparatus, such as is known for other environmental signals (Araújo et al., 2011).

Furthermore, the observed higher B accumulation by trees grafted on SW resulted from their ability to maintain canopy development through leaf production rather than leaf transpiration per unit of leaf area under varying B concentrations (**Figures 1A,C,D**), which in turn triggered high whole canopy transpiration. Taking into account plant leaf area (assuming an average specific leaf mass of 150 g m^-2^) and leaf transpiration values, we were able to estimate canopy transpiration. Such estimates revealed that the transpiration rate of trees on SW was almost 1.8-fold higher than that of trees on SK under B deficiency, i.e., 0.86 vs. 0.48 mmol s^-1^. This contribution of whole canopy transpiration to the B content in the plants is also supported by the fact that, despite the approximately 20% increase in DM of trees on SW with increasing B supply (**Figure 1**), total B accumulation in the same plants increased more than eightfold over the same supply range (**Figure 3A**). Accordingly, our preliminary results have shown that water transport in trees grafted on SW is higher than that of trees grafted on SK (unpublished data).

Plants grafted on SW presented lower photosynthetic rates per unit leaf area compared to those on SK under B deficiency (**Figure 4E**). However, the maintenance of leaf area likely compensated low photosynthetic rates of the former, promoting higher canopy photosynthesis.

As the stomata were open (**Figure 4E**), the carboxylation efficiency was not changed (**Figure 4F**) and there were no signals of damage to thylakoids (**Figures 8A,D**), we may argue that photosynthesis was down regulated by reduction of plant growth (sink activity) in plants grafted on SK and subjected to B deficiency. We already know that B-deficient citrus plants can accumulate carbohydrates in leaves (Han et al., 2009), which in turn can down-regulate photosynthesis (Hans et al., 2008; Ruuhola et al., 2011; Ribeiro et al., 2012).

### Excess of Boron and Citrus Performance as Affected by Rootstock

Boron excess limited plant growth of trees grafted on SK (**Figures 1A,B,D,F**). Such sensitivity was also found in *g*_s,_ *A*, and *k* (**Figures 4C,E,F**) and it is in accordance with the ultrastructural damage observed in stomata (**Figure 5L**) and chloroplasts (**Figure 8F**). Increased production of reactive oxygen species, thereby altering the structure and function of choroplasts (Hans et al., 2008), could explain the reduction of photosynthesis and plant growth on SK at 5.0 mg L^-1^ of B. Oxidative damage caused by excessive B supply has already been reported in less adaptive B-tolerant *Citrus* species, as characterized by a differential profile of proteins involved in antioxidant and detoxification systems in the leaves of plants under B excess (Sang et al., 2015). Plants grafted on SW showed little anatomical and ultrastructural damage and had leaf gas exchange and water relations less responsive to deficiency or excess of B when compared with those on SK. In addition, plants grafted on SW maintained higher growth, even when leaf B levels were considered high or excessive (Papadakis et al., 2004), which did not occur for those on SK. These results suggest that the interpretation of leaf chemical analysis in citrus plants should be revised and consider the rootstock, as our data indicate that the toxic B level in the leaves of trees grafted on SW might be higher than that of trees on SK. The underlying mechanisms of interaction and reciprocal signaling between scion and rootstock are complex and still poorly understood. In fact, the nutritional status of scions is significantly changed by rootstocks and the mechanisms related to compartmentalization and immobilization of nutrients in excess should be investigated, taking into account the scion/rootstock combination. The choose of SW as rootstock is a useful strategy for high- yielding orchards, which require improved management of B nutrition, as plants have shown enhanced ability to adjust their growth under changing B availability in rooting medium. Such variability in B supply is frequently found under field conditions due to nutrient leaching in tropical soils or accumulation in the solution of saline soils.

## Conclusion

Our results reveal that rootstocks influence citrus tree performance under varying B supply. The performance of Swingle rootstock under low B supply was characterized by greater accumulation of B in plants. This response was associated with the maintenance of a well-structured xylem system, which sustained water and B transport to the plant canopy and then leaf photosynthesis. Under high B supply, Swingle rootstock was still able to limit anatomical and ultrastructural damages and maintain water transport, preventing the negative impact of excessive B on plant growth.

## Author Contributions

GM, JQ, RB, FZ, and DM designed and performed the experiment, collected and analysed data; FT supervised microscopy analyses; RR supervised assessment of plant physiological traits; GM, RR, FZ, and DM critically revised and edited the final manuscript version. All authors discussed the results and commented on the manuscript.

## Conflict of Interest Statement

The authors declare that the research was conducted in the absence of any commercial or financial relationships that could be construed as a potential conflict of interest.

## Abbreviations

*A*: CO_2_ assimilation
*Ci*: intercellular CO_2_ concentration
DM: dry mass
DXV: diameter of xylem vessels
*E*: transpiration
g*_s_*: stomatal conductance
*k*: instantaneous carboxylation efficiency
*K*_L_: whole-plant leaf specific hydraulic conductance
LDM: dry mass of leaves
RD: root diameter
RDM: dry mass of roots
SDM: dry mass of shoots
SEM: scanning electron microscopy
SK: Sunki mandarin rootstock [*Citrus sunki* (Hayata) hort. ex Tanaka]
SW: Swingle citrumelo rootstock [*Citrus paradisi* Macf. × *Poncirus trifoliata* (L.) Raf.]
TEM: transmission electron microscopy
TLM: thickness of leaf mesophyll
TXCW: thickness of xylem cell wall
Ψ: leaf water potential
